# Clinical and ultrasound predictors of clinically significant endometrial pathology in women with histologically confirmed lesions: a retrospective case–control study

**DOI:** 10.3389/fonc.2026.1899603

**Published:** 2026-08-26

**Authors:** Xiao Yuhong, Wang Bolun, Wang Xiliang, Yan Li, Yu Tingting, Xiu Yinling, Yuexin Yu, Ma Xiangwei

**Affiliations:** Department of Reproductive Medicine, General Hospital of Northern Theater Command, Shenyang, Liaoning, China

**Keywords:** case-control study, clinicosonographic model, doppler ultrasound, EIN, endometrial cancer, endometrial pathology, prediction model, transvaginal ultrasound

## Abstract

**Objective:**

To identify clinical and ultrasound predictors of clinically significant endometrial pathology and to develop an internally validated clinicosonographic model in women with histologically confirmed endometrial or intracavitary lesions.

**Methods:**

This unmatched retrospective case–control study included women with histologically confirmed lesions who had complete clinicosonographic data. All eligible women with clinically significant pathology during the study period were included (n=500), and a random 1:1 sample of eligible benign-pathology controls was selected. The primary outcome was clinically significant pathology, defined as endometrial intraepithelial neoplasia (EIN)/atypical hyperplasia or endometrial carcinoma. Univariable and multivariable logistic regression analyses were performed to evaluate clinical and sonographic predictors. Three prespecified models were developed: clinical, sonographic, and combined clinicosonographic models. Model discrimination was assessed using the area under the receiver operating characteristic curve (AUC), and internal validation was performed with bootstrap resampling.

**Results:**

Compared with benign pathology, clinically significant pathology was associated with older age, lower parity, greater endometrial thickness, larger lesion size, mixed/heterogeneous echogenicity, abnormal endomyometrial junction, higher Color Doppler flow imaging [CDFI] score, and more frequent polypoid mass-like morphology, while the bright-edge sign was more common in benign pathology. In the final combined model, independent predictors of clinically significant pathology were age (adjusted odds ratio [aOR] per 5-year increase 11.49, 95% confidence interval [CI] 8.10–16.28), parity (aOR per additional birth 0.63, 95% CI 0.53–0.75), endometrial thickness (aOR per 5-mm increase 1.98, 95% CI 1.55–2.53), lesion length (aOR per 1-mm increase 1.52, 95% CI 1.29–1.80), mixed/heterogeneous echogenicity (aOR 1.87, 95% CI 1.13–3.09), abnormal endomyometrial junction (aOR 1.80, 95% CI 1.14–2.83), minimal/moderate/abundant CDFI score versus none, bright-edge sign (aOR 0.37, 95% CI 0.20–0.66), and polypoid mass-like lesion (aOR 3.85, 95% CI 2.42–6.12). The combined model showed the best discrimination, with an AUC of 0.959 and optimism-corrected AUC of 0.957.

**Conclusions:**

Clinically significant endometrial pathology is associated with a distinct clinicosonographic profile that includes older age, lower parity, thicker endometrium, larger lesions, heterogeneous echogenicity, junctional abnormality, increased vascularity, and polypoid mass-like morphology, whereas the bright-edge sign appears reassuring. A combined clinical and ultrasound model showed excellent internal discrimination and may help structure risk assessment in women with pathology-confirmed endometrial or intracavitary lesions.

## Introduction

Transvaginal ultrasonography is the first-line imaging test for evaluating the endometrium because it is widely available, noninvasive, and capable of capturing both endometrial thickness and lesion morphology (1, 2). In routine practice, however, decision-making is still often anchored to endometrial-thickness thresholds, especially in women with postmenopausal bleeding (1). Although that approach is clinically useful, it is incomplete: thickness alone does not fully capture focal intracavitary disease, vascular behavior, or the heterogeneity of lesions encountered in real-world gynecologic practice (2, 3). As a result, women with very different histopathologic diagnoses may enter the same diagnostic pathway despite having markedly different sonographic phenotypes and malignancy risk (2, 3).

A major step forward in this field was the International Endometrial Tumor Analysis (IETA) consensus, which standardized the terminology used to describe endometrial thickness, echogenicity, endometrial outline, endomyometrial junction, intracavitary fluid, CDFI score, and vascular patterns (2). Subsequent multicenter IETA studies showed that malignant and premalignant lesions differ from benign lesions not only in thickness, but also in qualitative grayscale and Doppler features, including heterogeneous echogenicity, abnormal junctional appearance, and richer vascularity, both in women with abnormal uterine bleeding and in women without bleeding (3–5). These studies have helped move endometrial ultrasonography beyond a single-threshold paradigm and toward structured phenotyping (2–5).

Prediction modeling has evolved in parallel with this descriptive work. The IETA-1 model represented an important advance by integrating multiple ultrasound features into a multinomial risk model in women with abnormal uterine bleeding (6). Later validation work showed that the model also discriminated reasonably well in women without abnormal uterine bleeding, although calibration was imperfect and risk was overestimated in some settings, underscoring that transportability cannot be assumed (7). In addition, several institutional models have been developed in narrower populations, such as postmenopausal women with bleeding and thickened endometrium, which limits generalizability across the broader spectrum of women referred for endometrial or intracavitary lesions (8).

Important gaps therefore remain. First, there is still a need for pathology-confirmed, clinically interpretable models that integrate both clinical and sonographic information rather than relying on endometrial thickness alone (6–8). Second, many prior studies have focused primarily on endometrial carcinoma, whereas clinical decision-making often hinges on the broader category of clinically significant pathology, including endometrial intraepithelial neoplasia (EIN)/atypical hyperplasia as well as carcinoma (6, 9). Third, although the IETA studies define the strongest international benchmark, local validation in routine institutional practice remains essential because referral patterns, pathology mix, and reporting habits can materially influence model behavior (6–8).

The present study was designed to identify which clinical and ultrasound features independently predict clinically significant endometrial pathology in a pathology-confirmed retrospective case–control study, and to develop an internally validated parsimonious clinicosonographic model for that purpose. By integrating age and reproductive history with lesion size, echogenicity, junctional abnormality, vascularity, and classic benign markers such as the bright-edge sign, this study aims to bridge the gap between standardized IETA-style sonographic assessment and practical risk estimation in women with endometrial or intracavitary lesions (2, 6, 10).

## Methods

### Study design and reporting framework

This was an unmatched retrospective case–control study designed to identify clinical and sonographic predictors of clinically significant endometrial pathology and to develop an internally validated clinicosonographic diagnostic prediction model. The report was prepared primarily in accordance with the TRIPOD+AI reporting framework for prediction-model studies and secondarily aligned with the STROBE case-control checklist for observational studies (11, 12). No machine-learning algorithm was used; the prediction model was developed using multivariable logistic regression.

### Study setting and data source

We conducted a retrospective review of women evaluated during the study period from 2015 to 2025 at department of Reproductive Medicine, General Hospital of Northern Theater Command, Shenyang, China who underwent pelvic ultrasound followed by histopathologic assessment of an endometrial or intracavitary lesion. Clinical, laboratory, ultrasound, procedural, and pathology data were extracted from the electronic medical record from the hospital information system and ultrasound reporting system using a prespecified case-report form.

### Participants

**Figure 1:**
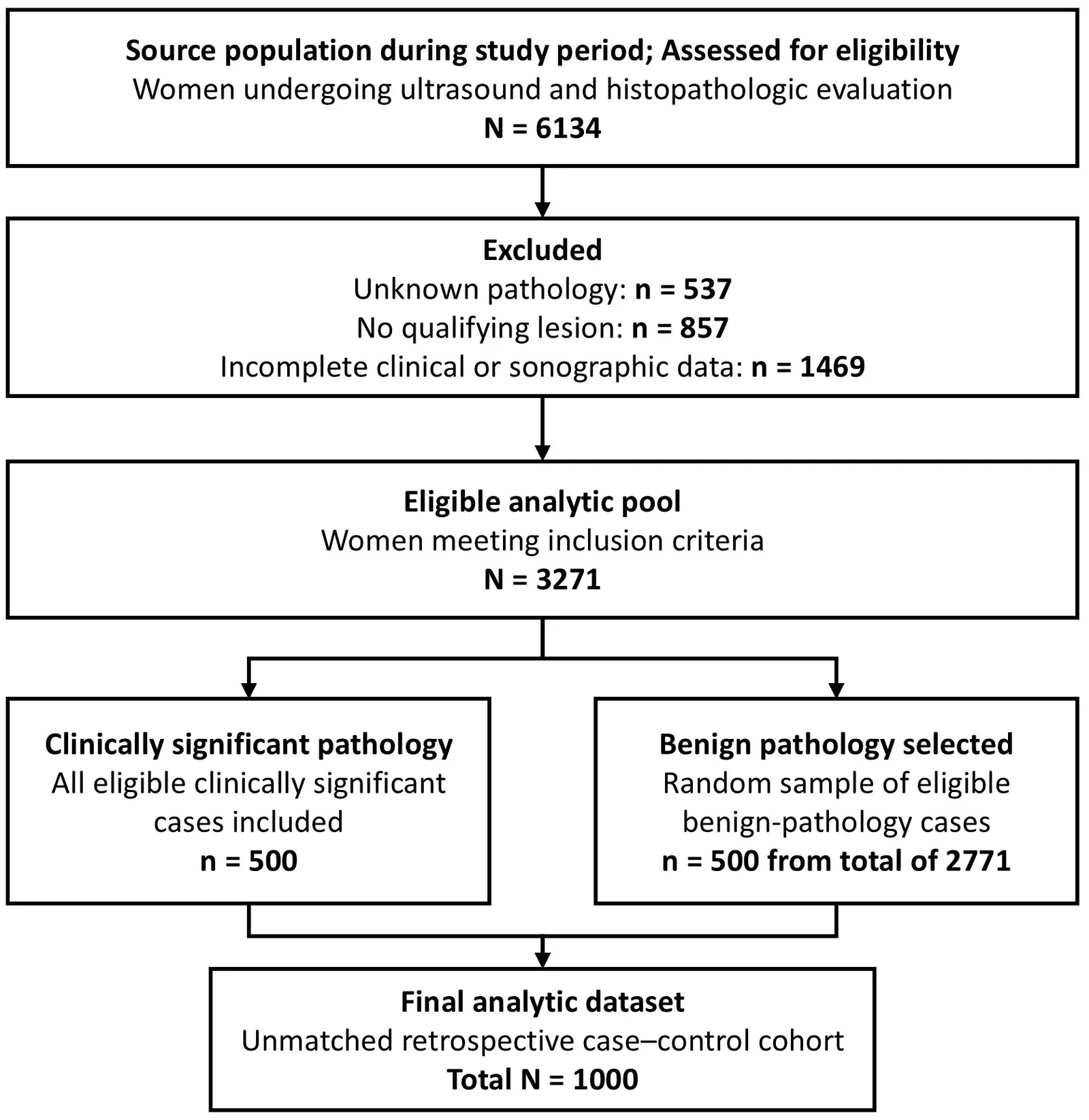
Study flow diagram of participant selection. Among 3271 eligible women with histologically confirmed endometrial or intracavitary pathology and complete core clinicosonographic data, 500 had clinically significant pathology and 2771 had benign pathology. All 500 clinically significant cases were included. From the 2771 eligible benign-pathology patients, 500 controls were randomly selected without replacement using a computer-generated random sequence in R; the remaining 2271 eligible benign-pathology patients were not included in the primary 1:1 case–control analytic sample. Women with no qualifying endometrial or intracavitary lesion were excluded because the study model was designed for lesion-based risk stratification and included lesion-level ultrasound predictors that were not applicable in lesion-free patients.

Women were eligible if they had histologically confirmed endometrial or intracavitary pathology; available complete pre-procedural clinicosonographic data; and complete data for the prespecified core predictors used in the main analyses including but not limited to age, parity, BMI, bleeding status, family history. Women were excluded if pathology was unknown, no qualifying lesion was present or essential clinicosonographic data were incomplete. From the eligible analytic pool, all women with clinically significant pathology were included. Eligible women with benign pathology constituted the control sampling frame. A computer-generated random sequence was created in R, and 500 benign-pathology controls were selected without replacement and without matching to create a 1:1 case–control analytic sample. The participant flow is shown in **Figure 1**.

The target population of the present model was restricted to women with a qualifying endometrial or intracavitary lesion because the main sonographic predictors were lesion-level characteristics, including lesion size, echogenicity, endomyometrial-junction appearance, Doppler vascularity, bright-edge sign, and polypoid mass-like morphology. Women without a qualifying lesion were therefore excluded from the main analytic sample because these lesion-level variables were not applicable. Including such patients would have changed the clinical question from lesion-based risk stratification to a broader screening model among all women undergoing ultrasound.

### Outcome definition

The primary outcome was clinically significant endometrial pathology, defined as histologically confirmed endometrial intraepithelial neoplasia (EIN)/atypical hyperplasia or endometrial carcinoma. The benign comparison group consisted of histologically confirmed benign pathology, including atrophy/normal endometrium (apart from the lesion), endometrial polyp, intracavitary myoma, hyperplasia without atypia/endometritis/benign proliferative-secretory endometrium, and other benign diagnoses. A secondary exploratory case-only comparison evaluated women with EIN/atypical hyperplasia versus women with endometrial carcinoma.

The reference standard was histopathologic diagnosis obtained through routine clinical care using one of the following procedures: hysteroscopic biopsy, dilation and curettage (D&C), polypectomy/resection, or hysterectomy. When multiple pathology reports were available, the final diagnosis was based on the most definitive histopathologic specimen from the diagnostic episode.

## Predictor variables

### Clinical variables

The following clinical and demographic variables were collected: age, age at menarche, age at menopause, years since menopause, menopausal status, gravidity, parity, body mass index (BMI), abnormal uterine bleeding type, lower abdominal pain, vaginal discharge, history of hysteroscopy, hypertension, diabetes, polycystic ovary syndrome or chronic anovulation, tamoxifen use, hormone replacement therapy, and family history of gynecologic cancer. Laboratory variables included hemoglobin and platelet count. For the main clinical model, the prespecified candidate predictors were age, parity, BMI, family history of gynecologic cancer, postmenopausal bleeding, history of hysteroscopy, tamoxifen use, and hormone replacement therapy.

### Sonographic variables

Ultrasound terminology and measurements were interpreted according to the International Endometrial Tumor Analysis (IETA) consensus, with reporting principles also aligned with the ISUOG consensus statement on endometrial sonographic assessment (2, 10). Ultrasound variables included endometrial thickness, lesion length, lesion width, lesion anteroposterior diameter, lesion volume, ultrasound route, primary echogenicity, echogenicity uniformity, endometrial outline, endometrial midline, intracavitary fluid, endomyometrial junction, Color Doppler flow imaging (CDFI) score, vascular pattern, lesion count, lesion location, bright-edge sign, blood-flow signal presence, and polypoid mass-like lesion. The prespecified sonographic candidate predictors for the main model include endometrial thickness, lesion length, mixed/heterogeneous primary echogenicity, abnormal endomyometrial junction, CDFI score, bright-edge sign, and polypoid mass-like lesion.

### Predictor measurement and coding

Ultrasound predictors were obtained from the pre-procedural ultrasound report interpreted by attending gynecologic sonographers with access to standard departmental reporting templates. All ultrasound examinations were performed as part of routine clinical care by attending gynecologic sonographers in the hospital ultrasound department. Reports were generated using departmental reporting templates that included standardized descriptors for endometrial thickness, lesion dimensions, echogenicity, endomyometrial junction status, Doppler vascularity, and lesion morphology. Therefore, formal interobserver agreement statistics were not available. Endometrial thickness and lesion dimensions were recorded in millimeters. Lesion volume was derived from the three orthogonal lesion diameters using the ellipsoid formula and expressed in milliliters. Menopausal status was classified as pre-menopausal, peri-menopausal, or post-menopausal, as recorded by the treating/visiting physician. Abnormal uterine bleeding was categorized as none, intermenstrual/irregular bleeding, heavy menstrual bleeding, postcoital/contact bleeding, postmenopausal bleeding, or mixed/other.

For regression analyses, postmenopausal bleeding was coded as a binary variable (yes/no). Mixed/heterogeneous primary echogenicity was coded as yes/no. Abnormal endomyometrial junction was defined as an irregular or interrupted junction. CDFI score was treated as an ordinal categorical predictor with no flow as the reference category. Bright-edge sign, blood-flow signal presence, and polypoid mass-like lesion were coded as binary variables. Age was modeled as a continuous predictor and scaled per 5-year increase by dividing age in years by 5 before entry into the logistic regression models. Age was not categorized, log-transformed, standardized, or otherwise transformed.

### Sample size

The final analytic dataset included 1000 women, comprising 500 women with clinically significant pathology and 500 women with benign pathology. All eligible clinically significant cases were included and benign controls were randomly sampled at a 1:1 ratio. Given the number of outcome events and the prespecified predictor set, the sample was considered adequate for the planned logistic-regression analyses.

### Statistical analysis

The primary outcome and the prespecified predictors used in the main models were complete in the final analytic dataset (No missing data). Therefore, the main analyses used a complete-case approach.

Normality of continuous variables was assessed using the Shapiro–Wilk test, together with visual inspection of histograms and Q–Q plots. Because the main continuous variables showed departures from normality in the overall analytic sample (**Supplementary Table S6**), continuous variables were summarized as median and interquartile range (IQR), and categorical variables as number and percentage. Between-group comparisons used the Mann–Whitney U test for continuous variables and the Pearson chi-square test for categorical variables. Fisher’s exact test was used when expected cell counts were small. Univariable binary logistic regression was first used to estimate crude odds ratios (ORs) and 95% confidence intervals (CIs) for prespecified clinical and sonographic predictors of clinically significant pathology.

Three prespecified multivariable logistic regression models were fitted including a clinical model, a sonographic model, and a combined parsimonious clinicosonographic model. The clinical model included age, parity, BMI, family history of gynecologic cancer, postmenopausal bleeding, history of hysteroscopy, tamoxifen use, and hormone replacement therapy. The sonographic model included endometrial thickness, lesion length, mixed/heterogeneous primary echogenicity, abnormal endomyometrial junction, CDFI score, bright-edge sign, and polypoid mass-like lesion. The combined parsimonious model included age, parity, endometrial thickness, lesion length, mixed/heterogeneous primary echogenicity, abnormal endomyometrial junction, CDFI score, bright-edge sign, and polypoid mass-like lesion. Predictors were selected *a priori* based on clinical plausibility, interpretability, and avoidance of redundancy. To reduce collinearity, the binary blood-flow-signal variable was not entered together with CDFI score in the main models. Continuous predictors were retained on clinically interpretable scales: age per 5-year increase, endometrial thickness per 5-mm increase, and lesion length per 1-mm increase.

Model discrimination was assessed using the area under the receiver operating characteristic curve (AUC). For descriptive comparison, sensitivity and specificity were estimated at the Youden index-derived cutoff. Internal validation was performed using bootstrap resampling with 120 bootstrap samples, and optimism-corrected AUC and optimism-corrected calibration slope were reported. Because the final analytic dataset used 1:1 case-control sampling, model discrimination and calibration were interpreted within the selected analytic sample. The sampling fraction was not prevalence-preserving; therefore, predicted probabilities from the logistic models should not be interpreted as absolute clinical risk without recalibration in a representative target population.

Several prespecified supplementary analyses were performed to assess robustness, including a calendar-year adjusted model (with year added as a fixed-effect covariate); a model excluding women with benign atrophy/normal histology; a model restricted to postmenopausal women; and a carcinoma-only outcome analysis, excluding EIN/atypical hyperplasia. An additional exploratory comparison was conducted within the clinically significant group to compare women with EIN/atypical hyperplasia versus women with endometrial carcinoma.

To assess whether random benign-control subsampling materially affected model discrimination, an additional sensitivity analysis was performed using the full eligible benign cohort. In this analysis, the same combined clinicosonographic model was fitted using all 500 clinically significant cases and all 2771 eligible benign-pathology patients.

All statistical analyses were performed using R version 4.4.3. Forest plot was drawn using GraphPad Prism, version 9.

### Ethical considerations

The study was reviewed and approved by the Ethics Committee of General Hospital of Northern Theater Command under approval number [Y (2015) 046]. Because of the retrospective design and use of routinely collected clinical data, the requirement for informed consent was waived. All data were analyzed in de-identified form in accordance with the Declaration of Helsinki.

## Results

### Study population and pathology spectrum

During the study period, 6134 women undergoing ultrasound and histopathologic evaluation were assessed for eligibility. After exclusion of women with unknown pathology, no qualifying lesion, or incomplete clinicosonographic data, 3271 remained in the eligible analytic pool. All 500 eligible women with clinically significant pathology were included, and 500 women with benign pathology were randomly selected, yielding a final unmatched retrospective case–control study of 1000 women (**Figure 1**). Within the clinically significant group, 240 women (48.0%) had EIN/atypical hyperplasia and 260 (52.0%) had endometrial carcinoma. Within the benign group, the most common diagnoses were atrophy/normal histology (47.8%) and endometrial polyp (32.2%) (**Supplementary Table 1**).

### Baseline clinical characteristics

Baseline clinical characteristics are summarized in **Table 1**. Compared with women with benign pathology, those with clinically significant pathology were older (median 61 vs 53 years, *P* < 0.001), had lower parity (median 2 vs 3, *P* < 0.001), and had slightly higher BMI (median 23.1 vs 22.7 kg/m², *P* = 0.016). Hemoglobin was lower in the clinically significant group (13.2 vs 14.5 g/dL, *P* < 0.001), whereas platelet count was higher (316 vs 273 ×10^9^/L, *P* < 0.001). Menopausal status differed markedly between groups, with postmenopausal women comprising 94.8% of the clinically significant group compared with 46.2% of the benign group (*P* < 0.001). Postmenopausal bleeding was more frequent among women with clinically significant pathology (32.2% vs 18.0%), while intermenstrual/irregular bleeding and heavy menstrual bleeding were more common in the benign group. History of hysteroscopy, tamoxifen use, hormone replacement therapy, and family history of gynecologic cancer were also more frequent in the clinically significant group (all *P ≤* 0.016) (**Table 1**).

**Table 1:** Baseline clinical characteristics according to final pathology status.

Variable	Benign pathology (n=500)	Clinically significant pathology (n=500)	Overall (N = 1000)	P value
Age, years	53 (50–56)	61 (58–63)	57 (52–61)	<0.001
Parity	3 (2–4)	2 (1–3)	3 (2–4)	<0.001
BMI, kg/m²	22.7 (21.3–24.2)	23.1 (21.6–24.5)	22.9 (21.4–24.5)	0.016
Hemoglobin, g/dL	14.5 (14.0–15.1)	13.2 (12.8–13.7)	13.9 (13.2–14.6)	<0.001
Platelet count, ×10^9^/L	273 (184–356)	316 (224–406)	296 (203–383)	<0.001
**Menopausal status, n (%)**				<0.001
Pre-menopausal	142 (28.4)	6 (1.2)	148 (14.8)	
Peri-menopausal	127 (25.4)	20 (4.0)	147 (14.7)	
Post-menopausal	231 (46.2)	474 (94.8)	705 (70.5)	
**Abnormal uterine bleeding type, n (%)**				<0.001
None	176 (35.2)	175 (35.0)	351 (35.1)	
Intermenstrual/irregular bleeding	53 (10.6)	6 (1.2)	59 (5.9)	
Heavy menstrual bleeding	56 (11.2)	2 (0.4)	58 (5.8)	
Postcoital/contact bleeding	91 (18.2)	109 (21.8)	200 (20.0)	
Postmenopausal bleeding	90 (18.0)	161 (32.2)	251 (25.1)	
Mixed/other	34 (6.8)	47 (9.4)	81 (8.1)	
History of hysteroscopy, yes	239 (47.8)	306 (61.2)	545 (54.5)	<0.001
Tamoxifen use, yes	15 (3.0)	31 (6.2)	46 (4.6)	0.016
Hormone replacement therapy, yes	20 (4.0)	43 (8.6)	63 (6.3)	0.003
Family history of gynecologic cancer, yes	19 (3.8)	59 (11.8)	78 (7.8)	<0.001

Continuous variables are presented as median (IQR) and categorical variables as n (%). P values represent between-group comparisons between women with benign pathology and women with clinically significant endometrial pathology. Continuous variables were compared using the Mann–Whitney U test; categorical variables were compared using the Pearson chi-square test or Fisher’s exact test, as appropriate; BMI, body mass index; IQR, interquartile range. Bold text indicates the main categorical variable/group heading; it does not denote statistical significance.

### Sonographic characteristics

Key sonographic characteristics are shown in **Table 2**. Women with clinically significant pathology had greater endometrial thickness than those with benign pathology (median 12.0 vs 9.4 mm, *P* < 0.001). Lesion dimensions were also larger in the clinically significant group, including lesion length (10.6 vs 9.7 mm), lesion width (8.6 vs 7.9 mm), anteroposterior diameter (7.5 vs 6.7 mm), and lesion volume (0.4 vs 0.3 mL) (all *P* < 0.001). The clinically significant group more often had multiple lesions, with a median lesion count of 2 compared with 1 in the benign group (*P* < 0.001).

**Table 2:** Key sonographic characteristics according to final pathology status.

Variable	Benign pathology (n=500)	Clinically significant pathology (n=500)	Overall (N = 1000)	P value
Endometrial thickness, mm	9.4 (6.0–12.8)	12.0 (8.8–15.2)	10.7 (7.5–14.0)	<0.001
Lesion length, mm*	9.7 (8.7–10.6)	10.6 (9.6–11.5)	10.1 (9.1–11.2)	<0.001
Lesion width, mm	7.9 (7.3–8.5)	8.6 (8.0–9.3)	8.3 (7.5–9.0)	<0.001
Lesion anteroposterior diameter, mm	6.7 (6.0–7.3)	7.5 (6.7–8.2)	7.0 (6.3–7.8)	<0.001
Lesion volume, mL	0.3 (0.2–0.3)	0.4 (0.3–0.4)	0.3 (0.2–0.4)	<0.001
Number of lesions	1 (1–2)	2 (1–2)	2 (1–2)	<0.001
**Primary echogenicity, n (%)**				<0.001
Hypoechoic	37 (7.4)	21 (4.2)	58 (5.8)	
Isoechoic	140 (28.0)	86 (17.2)	226 (22.6)	
Hyperechoic	191 (38.2)	171 (34.2)	362 (36.2)	
Mixed/heterogeneous	102 (20.4)	177 (35.4)	279 (27.9)	
Three-layer pattern	29 (5.8)	37 (7.4)	66 (6.6)	
Not assessable	1 (0.2)	8 (1.6)	9 (0.9)	
**Endometrial outline, n (%)**				<0.001
Smooth	271 (54.2)	220 (44)	491 (49.1)	
Irregular	101 (20.2)	207 (41.4)	308 (30.8)	
Bright edge present	123 (24.6)	53 (10.6)	176 (17.6)	
Not assessable	5 (1)	20 (4)	25 (2.5)	
**Endomyometrial junction, n (%)**				<0.001
Regular	327 (65.4)	260 (52.0)	587 (58.7)	
Irregular	135 (27.0)	175 (35.0)	310 (31.0)	
Interrupted	30 (6.0)	58 (11.6)	88 (8.8)	
Not defined	7 (1.4)	7 (1.4)	14 (1.4)	
Not assessable	1 (0.2)	0 (0.0)	1 (0.1)	
**CDFI score, n (%)**				<0.001
None	242 (48.4)	133 (26.6)	375 (37.5)	
Minimal	172 (34.4)	196 (39.2)	368 (36.8)	
Moderate	70 (14.0)	142 (28.4)	212 (21.2)	
Abundant	16 (3.2)	29 (5.8)	45 (4.5)	
Bright-edge sign, yes	123 (24.6)	53 (10.6)	176 (17.6)	<0.001
Blood-flow signal present, yes	263 (52.6)	333 (66.6)	596 (59.6)	<0.001
Polypoid mass-like lesion, yes	105 (21.0)	281 (56.2)	386 (38.6)	<0.001

Continuous variables are presented as median (IQR) and categorical variables as n (%). P values represent between-group comparisons between women with benign pathology and women with clinically significant endometrial pathology. Continuous variables were compared using the Mann–Whitney U test; categorical variables were compared using the Pearson chi-square test or Fisher’s exact test, as appropriate; CDFI, Color Doppler flow imaging; IQR, interquartile range. Bold text indicates the main categorical variable/group heading; it does not denote statistical significance.

Marked differences were also observed in qualitative ultrasound features. Mixed/heterogeneous echogenicity was more common in clinically significant pathology than in benign pathology (35.4% vs 20.4%), whereas isoechogenic lesions were more common in benign pathology (28.0% vs 17.2%) (*P* < 0.001 overall). Endometrial outline differed substantially between groups: smooth outline was more frequent in benign pathology (54.2% vs 44.0%), while irregular outline was more frequent in clinically significant pathology (41.4% vs 20.2%); by contrast, a bright-edge outline was more common in benign pathology (24.6% vs 10.6%) (*P* < 0.001 overall). Abnormal endomyometrial-junction features were also more frequent among women with clinically significant pathology, including both irregular and interrupted junctions (*P* < 0.001 overall). In addition, clinically significant lesions showed higher vascularity, with a shift toward moderate and abundant CDFI scores, more frequent detectable blood-flow signals (66.6% vs 52.6%, *P* < 0.001), and a markedly higher frequency of polypoid mass-like lesions (56.2% vs 21.0%, *P* < 0.001) (**Table 2**).

### Univariable predictors of clinically significant pathology

In univariable logistic regression (**Table 3**), older age was strongly associated with clinically significant pathology (OR per 5-year increase 10.76, 95% CI 8.10–14.30, *P* < 0.001). Lower parity was inversely associated with the outcome (OR per additional birth 0.65, 95% CI 0.59–0.72, *P* < 0.001). Other significant crude clinical predictors included BMI, postmenopausal bleeding, and family history of gynecologic cancer. Among sonographic variables, greater endometrial thickness, greater lesion length, mixed/heterogeneous primary echogenicity, abnormal endomyometrial junction, detectable blood-flow signal, and polypoid mass-like lesion were all associated with higher odds of clinically significant pathology, whereas the bright-edge sign was inversely associated with the outcome (OR 0.36, 95% CI 0.26–0.52, *P* < 0.001) (**Table 3**).

**Table 3:** Univariable logistic regression analysis of core clinical and ultrasound predictors for clinically significant endometrial pathology.

Predictor (reference)	Crude OR (95% CI)	P value
Age (Per 5-year increase)	10.76 (8.10–14.30)	<0.001
Parity (Per 1 birth increase)	0.65 (0.59–0.72)	<0.001
BMI (Per 1 kg/m² increase)	1.07 (1.01–1.14)	0.018
Postmenopausal bleeding (Yes vs no)	2.16 (1.61–2.91)	<0.001
Family history of gynecologic cancer (Yes vs no)	3.39 (1.99–5.77)	<0.001
Endometrial thickness (Per 5-mm increase)	1.83 (1.58–2.12)	<0.001
Lesion length (Per 1-mm increase)	1.55 (1.41–1.71)	<0.001
Mixed/heterogeneous primary echogenicity (Yes vs no)	2.14 (1.61–2.84)	<0.001
Abnormal endomyometrial junction^†^ (Yes vs no)	1.77 (1.37–2.29)	<0.001
Bright-edge sign (Yes vs no)	0.36 (0.26–0.52)	<0.001
Blood-flow signal present (Yes vs no)	1.80 (1.39–2.32)	<0.001
Polypoid mass-like lesion (Yes vs no)	4.83 (3.65–6.38)	<0.001

Univariable associations were estimated using binary logistic regression with clinically significant pathology as the dependent variable; CI, confidence interval; OR, odds ratio.

^†^ Abnormal endomyometrial junction was defined as irregular or interrupted.

## Multivariable models

### Clinical model

In the multivariable clinical model (**Table 4**), older age remained the strongest independent clinical predictor of clinically significant pathology (adjusted OR [aOR] per 5-year increase 10.81, 95% CI 7.99–14.62, *P* < 0.001). Lower parity also remained independently associated with clinically significant pathology (aOR per additional birth 0.64, 95% CI 0.55–0.74, *P* < 0.001). BMI and family history of gynecologic cancer remained significant, whereas postmenopausal bleeding, history of hysteroscopy, tamoxifen use, and hormone replacement therapy were no longer independently associated with the outcome after adjustment (**Table 4**).

**Table 4:** Multivariable logistic regression models evaluating clinical predictors of clinically significant endometrial pathology.

Predictor (reference)	Adjusted OR (95% CI)	Coefficient test	P value
Age (Per 5-year increase)	10.81 (7.99–14.62)	Wald z test	<0.001
Parity (Per 1 birth increase)	0.64 (0.55–0.74)	Wald z test	<0.001
BMI (Per 1 kg/m² increase)	1.11 (1.01–1.21)	Wald z test	0.023
Family history of gynecologic cancer (Yes vs no)	3.73 (1.48–9.42)	Wald z test	0.005
Postmenopausal bleeding (Yes vs no)	1.09 (0.71–1.66)	Wald z test	0.701
History of hysteroscopy (Yes vs no)	1.34 (0.91–1.98)	Wald z test	0.135
Tamoxifen use (Yes vs no)	1.70 (0.66–4.41)	Wald z test	0.274
Hormone replacement therapy (Yes vs no)	1.79 (0.80–4.03)	Wald z test	0.159

The model was fitted using multivariable binary logistic regression with clinically significant pathology as the dependent variable; reported P values are from the Wald z test; CI, confidence interval; OR, odds ratio.

### Sonographic model

In the multivariable sonographic model (**Table 5**), endometrial thickness (aOR per 5-mm increase 1.98, 95% CI 1.66–2.35, *P* < 0.001) and lesion length (aOR per 1-mm increase 1.58, 95% CI 1.41–1.77, *P* < 0.001) were independently associated with clinically significant pathology. Mixed/heterogeneous primary echogenicity and abnormal endomyometrial junction also remained significant. Increasing CDFI score was independently associated with higher odds of clinically significant pathology, with the strongest association observed for moderate CDFI score versus no flow (aOR 4.44, 95% CI 2.90–6.80, *P* < 0.001). The bright-edge sign remained inversely associated with clinically significant pathology (aOR 0.37, 95% CI 0.24–0.56, *P* < 0.001), whereas polypoid mass-like lesion remained a strong positive predictor (aOR 4.23, 95% CI 3.08–5.81, *P* < 0.001) (**Table 5**).

**Table 5:** Multivariable logistic regression models evaluating sonographic predictors of clinically significant endometrial pathology.

Predictor (reference)	Adjusted OR (95% CI)	P value
Endometrial thickness (Per 5-mm increase)	1.98 (1.66–2.35)	<0.001
Lesion length (Per 1-mm increase)	1.58 (1.41–1.77)	<0.001
Mixed/heterogeneous primary echogenicity (Yes vs no)	1.86 (1.32–2.61)	<0.001
Abnormal endomyometrial junction (Yes vs no)	1.65 (1.21–2.25)	0.002
CDFI score = minimal (vs none)	2.19 (1.54–3.11)	<0.001
CDFI score = moderate (vs none)	4.44 (2.90–6.80)	<0.001
CDFI score = abundant (vs none)	3.24 (1.52–6.88)	0.002
Bright-edge sign (Yes vs no)	0.37 (0.24–0.56)	<0.001
Polypoid mass-like lesion (Yes vs no)	4.23 (3.08–5.81)	<0.001

The model was fitted using multivariable binary logistic regression with clinically significant pathology as the dependent variable; reported P values are from the Wald z test; CI, confidence interval; OR, odds ratio.

### Combined parsimonious clinicosonographic model

**Figure 2:**
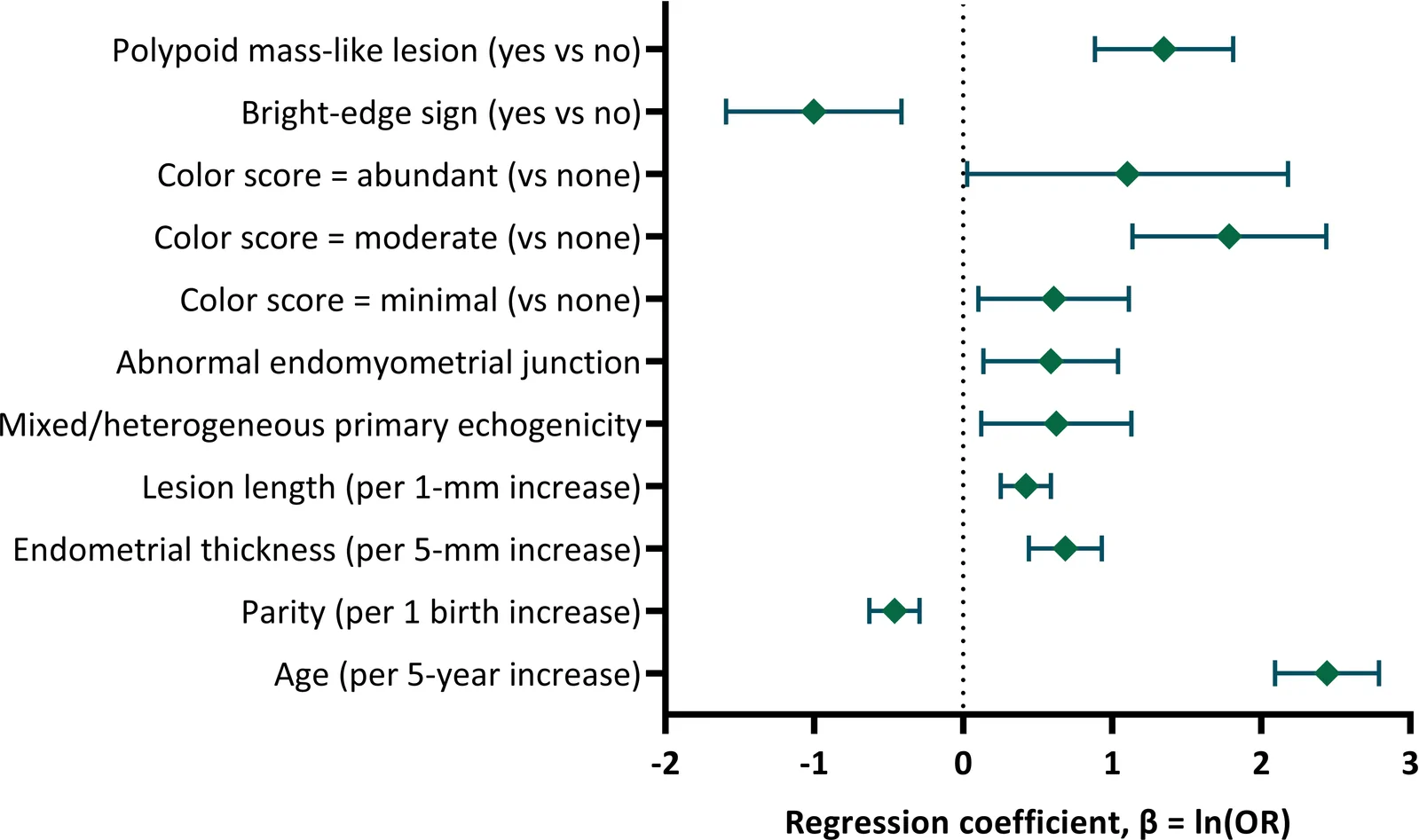
Forest plot of adjusted log odds ratios from the final combined clinicosonographic model, showing adjusted log odds ratios and 95% confidence intervals for each predictor.

In the final parsimonious combined model (**Table 6**), both clinical and sonographic variables contributed independent information. Older age remained the strongest independent predictor of clinically significant pathology. Age was entered as a continuous variable scaled per 5-year increase, yielding a regression coefficient of β=2.441 (SE = 0.178; 95% CI 2.092–2.790), corresponding to an aOR of 11.49 per 5-year increase (95% CI 8.10–16.28; P<0.001). Expressed per 1-year increase, this corresponds to an OR of approximately 1.63. Lower parity also remained independently associated with clinically significant pathology (aOR per additional birth 0.63, 95% CI 0.53–0.75, *P* < 0.001). Among ultrasound variables, greater endometrial thickness (aOR per 5-mm increase 1.98, 95% CI 1.55–2.53, *P* < 0.001), greater lesion length (aOR per 1-mm increase 1.52, 95% CI 1.29–1.80, *P* < 0.001), mixed/heterogeneous echogenicity (aOR 1.87, 95% CI 1.13–3.09, *P* = 0.015), and abnormal endomyometrial junction (aOR 1.80, 95% CI 1.14–2.83, *P* = 0.011) remained independently associated with clinically significant pathology. Compared with lesions without detectable vascularity, lesions with minimal, moderate, and abundant CDFI scores had progressively higher adjusted odds of clinically significant pathology, with the strongest effect seen for moderate CDFI score (aOR 5.97, 95% CI 3.11–11.43, *P* < 0.001). The bright-edge sign remained independently associated with lower odds of clinically significant pathology (aOR 0.37, 95% CI 0.20–0.66, *P* < 0.001), whereas polypoid mass-like lesion remained strongly associated with higher odds (aOR 3.85, 95% CI 2.42–6.12, *P* < 0.001) (**Table 6**; **Figure 2**).

**Table 6:** Multivariable logistic regression models evaluating combined parsimonious clinicosonographic predictors of clinically significant endometrial pathology.

Predictor (reference)	Adjusted OR (95% CI)	P value
Age (Per 5-year increase)	11.49 (8.10–16.28)	<0.001
Parity (Per 1 birth increase)	0.63 (0.53–0.75)	<0.001
Endometrial thickness (Per 5-mm increase)	1.98 (1.55–2.53)	<0.001
Lesion length (Per 1-mm increase)	1.52 (1.29–1.80)	<0.001
Mixed/heterogeneous primary echogenicity (Yes vs no)	1.87 (1.13–3.09)	0.015
Abnormal endomyometrial junction (Yes vs no)	1.80 (1.14–2.83)	0.011
CDFI score = minimal (vs none)	1.84 (1.11–3.04)	0.019
CDFI score = moderate (vs none)	5.97 (3.11–11.43)	<0.001
CDFI score = abundant (vs none)	3.01 (1.03–8.85)	0.045
Bright-edge sign (Yes vs no)	0.37 (0.20–0.66)	<0.001
Polypoid mass-like lesion (Yes vs no)	3.85 (2.42–6.12)	<0.001

The model was fitted using multivariable binary logistic regression with clinically significant pathology as the dependent variable; Age was modeled as a continuous variable scaled per 5-year increase, not categorized or transformed. Reported P values are from the Wald z test; CI, confidence interval; OR, odds ratio.

### Model discrimination and internal validation

**Figure 3:**
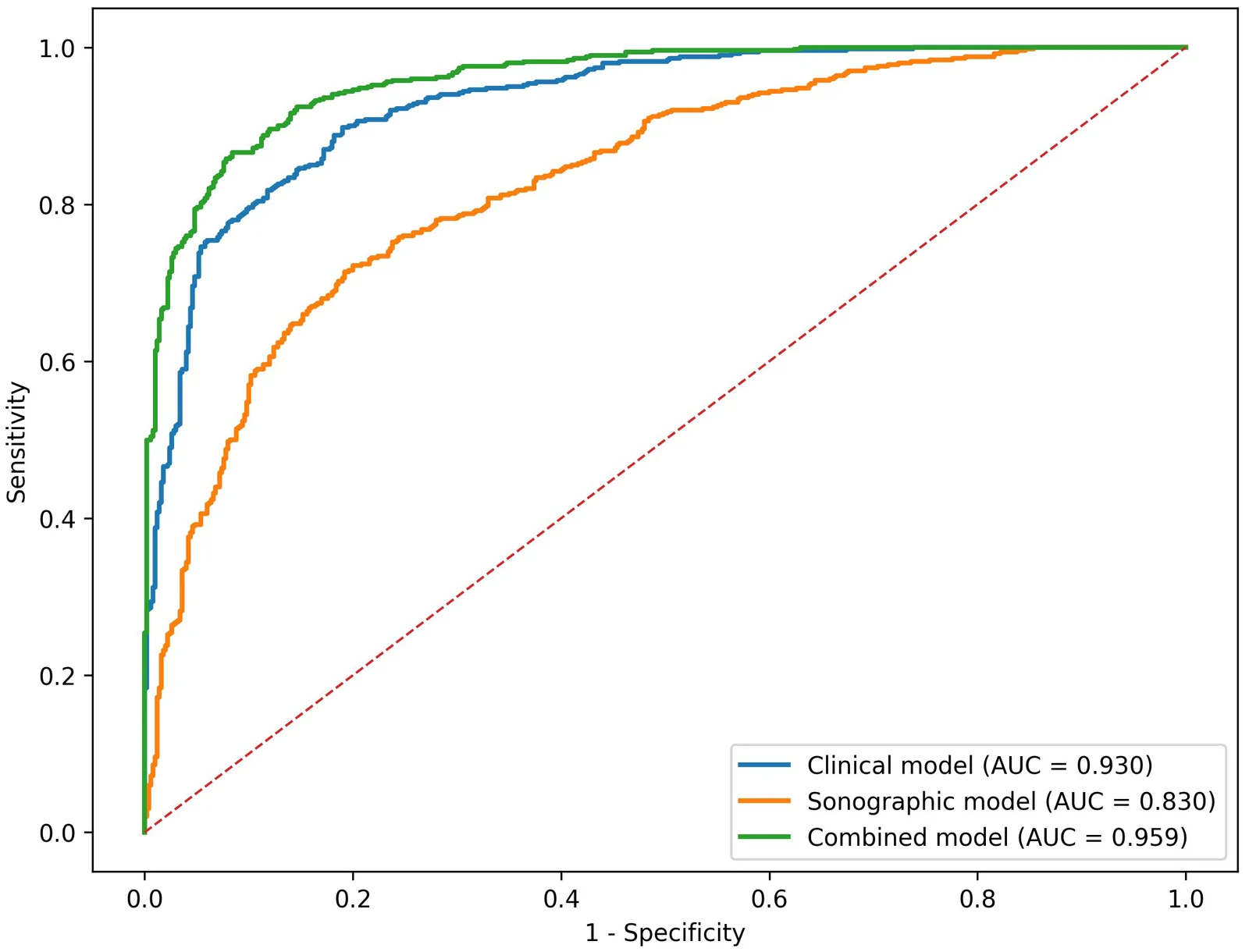
Receiver operating characteristic (ROC) curves for the clinical, sonographic, and combined clinicosonographic models.

Model performance is summarized in **Table 7** and visualized in **Figure 3**. The clinical model showed strong discrimination, with an AUC of 0.930 and an optimism-corrected AUC of 0.929. The sonographic model showed lower but still acceptable discrimination, with an AUC of 0.830 and an optimism-corrected AUC of 0.825. Within the selected 1:1 case-control analytic dataset, the combined clinicosonographic model achieved the best overall performance, with an AUC of 0.959, optimism-corrected AUC of 0.957, and optimism-corrected calibration slope of 0.962. At the Youden-index cutoff, the combined model yielded sensitivity of 0.862 and specificity of 0.916, outperforming both the clinical and sonographic models alone (**Table 7**; **Figure 3**).

**Table 7:** Discrimination and internal validation of the clinical, sonographic, and combined clinicosonographic models.

Model	No. of predictor families*	LR χ² test	P value	AIC	McFadden pseudo-R²	AUC	Optimism-corrected AUC^†^	Optimism-corrected calibration slope^†^	Sensitivity^‡^	Specificity^‡^
Clinical model	8	715.50	<0.001	688.79	0.516	0.930	0.929	0.980	0.898	0.810
Sonographic model	7	374.72	<0.001	1031.58	0.270	0.830	0.825	0.972	0.714	0.808
Combined clinicosonographic model	9	866.51	<0.001	543.78	0.625	0.959	0.957	0.962	0.862	0.916

AIC, Akaike information criterion; AUC, area under the receiver operating characteristic curve; LR χ², likelihood-ratio chi-square.

^*^Predictor families count grouped categorical variables such as CDFI score as one predictor family.

^†^Optimism-corrected AUC and optimism-corrected calibration slope were estimated by bootstrap internal validation using 120 bootstrap resamples.

^‡^Sensitivity and specificity were estimated by ROC analysis at the Youden-index cutoff within the analytic dataset.

### Supplementary analyses

The pathology spectrum of the analytic study is detailed in **Supplementary Table 1**, while the distribution of reference-standard procedures and ultrasound-to-pathology intervals is shown in **Supplementary Table 2**. Exploratory comparison within the clinically significant group showed no major differences between EIN/atypical hyperplasia and endometrial carcinoma for age, BMI, endometrial thickness, lesion length, lesion volume, CDFI score, polypoid morphology, or family history of gynecologic cancer, although hemoglobin was slightly lower in carcinoma (**Supplementary Table 3**). Calendar-year distribution is presented in **Supplementary Table 4**, and the main clinicosonographic findings remained robust in the prespecified sensitivity analyses, including calendar-year adjustment, exclusion of benign atrophy/normal histology, restriction to postmenopausal women, and carcinoma-only outcome analysis. In the sensitivity analysis using the full eligible benign cohort, the combined clinicosonographic model showed [AUC = 0.942], compared with [AUC = 0.959] in the primary 1:1 case–control analytic sample, suggesting that random benign-control subsampling did not materially affect model discrimination. (**Supplementary Table 5**).

## Discussion

In this pathology-confirmed retrospective case–control study, several findings emerged consistently across the descriptive analyses and multivariable models. Clinically significant endometrial pathology, defined as histologically confirmed endometrial intraepithelial neoplasia (EIN)/atypical hyperplasia or endometrial carcinoma, was associated with older age, lower parity, greater endometrial thickness, larger lesion size, mixed/heterogeneous echogenicity, abnormal endomyometrial junction, higher CDFI score, and polypoid mass-like morphology, whereas the bright-edge sign was independently associated with lower odds of clinically significant pathology. The combined clinicosonographic model outperformed the clinical-only and sonographic-only models, suggesting that the most clinically useful interpretation of endometrial risk is achieved when demographic/reproductive context and structured ultrasound morphology are considered together rather than in isolation.

The clinical component of our findings is broadly compatible with the established epidemiology of endometrial neoplasia. Older age and postmenopausal status are expected predictors of malignant and premalignant disease, while lower parity is a well-described risk pattern in endometrial cancer epidemiology (1, 13). Our results also align with more recent lesion-focused studies showing that menopause, abnormal uterine bleeding, obesity, and nulliparity increase the likelihood of premalignant or malignant endometrial pathology, particularly among women with polyps or other intracavitary lesions (8, 14). Nevertheless, the magnitude of the age coefficient should be interpreted cautiously. The large aOR likely reflects the strong age separation between clinically significant and benign pathology in this selected pathology-confirmed case–control dataset. In particular, the clinically significant group was substantially older and predominantly postmenopausal. Therefore, the age effect should be understood as a prediction-model coefficient within this restricted sampling frame, and external validation and recalibration would be necessary before using the model to estimate absolute risk in unselected clinical populations.

The sonographic findings are strongly concordant with the IETA literature. In both bleeding and non-bleeding populations, IETA studies have shown that malignant and premalignant lesions are more likely to demonstrate increased endometrial thickness, heterogeneous echogenicity, abnormal or interrupted junctional appearance, and richer vascularity, whereas benign lesions more often show more orderly morphology (3–5). Our study extends that pattern into a pathology-confirmed institutional study and shows that these features remain informative even when entered together with key clinical variables. The persistence of CDFI score and junctional abnormality in the final model is also consistent with prior work showing that grayscale and Doppler features track with tumor burden, grade, and biologic aggressiveness in endometrial cancer (5).

One of the more interesting aspects of the present study is the coexistence of two apparently opposing observations: the bright-edge sign behaved as a benign marker, while polypoid mass-like morphology was associated with clinically significant pathology. We do not view these results as contradictory. The bright-edge sign has long been considered a classic marker of endometrial polyps (15). By contrast, a polypoid contour alone is not synonymous with harmless disease. Recent studies of endometrial polyps show that the risk of premalignancy or malignancy rises in the presence of menopause, abnormal uterine bleeding, obesity, nulliparity, multiple polyps, and thickened endometrium (14). Accordingly, our findings suggest that a “polypoid” lesion should not automatically reassure the clinician when it coexists with suspicious echotexture or vascularity (14, 15).

The performance of the combined model should be interpreted in the context of the sampling design. The final analytic dataset used a 1:1 case-control structure, with all eligible clinically significant cases included and a random sample of benign-pathology controls selected from the eligible benign pool. This design is efficient for identifying predictors and estimating odds-ratio associations, but it is not prevalence-preserving. In the final analytic dataset, the clinically significant pathology fraction was 50%, whereas in the eligible lesion-confirmed analytic pool it was approximately 15.3% (500/3271). Therefore, the fitted intercept and predicted probabilities would overestimate absolute risk if applied directly to routine clinical practice (6–8).

Although AUC is a rank-based measure and is not determined by prevalence alone, the selected case-control structure and pathology-confirmed lesion-based sampling may have increased apparent separation between groups compared with an unselected population. Accordingly, the high AUC should be viewed as evidence of strong internal discrimination within this selected sample, not as proof of equivalent real-world diagnostic performance (6–8, 11). Before clinical implementation, the model would require external validation in a prevalence-preserving cohort and recalibration, particularly recalibration of the intercept and possibly the calibration slope, to match the baseline risk and predictor distribution of the target population.

Another result that deserves careful discussion is the lack of major separation between EIN/atypical hyperplasia and endometrial carcinoma in the exploratory case-only analysis. Rather than undermining the model, this finding is clinically plausible. Distinguishing EIN/AEH from carcinoma remains difficult even with modern imaging, and recent studies continue to show that concurrent carcinoma may be present in a substantial proportion of women with atypical endometrial hyperplasia (9). Our results therefore support the pragmatic decision to use a composite endpoint of clinically significant pathology rather than overinterpreting routine sonographic features as sufficient to distinguish premalignancy from established carcinoma (6, 9).

This study has several strengths. First, all analyses were anchored to a histopathologic reference standard rather than to imaging follow-up alone. Second, the dataset included a broad range of structured sonographic variables, allowing assessment of morphology and vascularity alongside thickness rather than reducing ultrasound to a single-threshold test. Third, the model was deliberately kept parsimonious and clinically interpretable, which is an advantage over more complex tools (11). Fourth, the sensitivity analyses were directionally reassuring, with core associations persisting after calendar-year adjustment, exclusion of benign atrophy/normal histology, restriction to postmenopausal women, and carcinoma-only outcome analysis.

The limitations are equally important. Most importantly, the case–control design allows inference about association, not causation, and it does not support direct estimation of source-population prevalence or absolute clinical risk. In addition, because benign controls were subsampled and the event fraction was artificially fixed at 50%, the model should not be used as an absolute-risk calculator without recalibration in a representative clinical cohort. Second, the study was retrospective and based on routine clinical reporting, which introduces the possibility of variability in ultrasound acquisition, interpretation, and registration across years and operators. Third, the data come from a single institutional setting and have not undergone external validation; accordingly, the high internal performance may not reproduce in other hospitals with different referral patterns or reporting conventions. Finally, although the clinically significant group combined EIN/AEH and carcinoma, that composite endpoint is arguably the most clinically relevant one because both entities usually trigger more definitive diagnostic or therapeutic action than benign pathology (6, 9).

Another limitation concerns the reproducibility of qualitative ultrasound predictors. Several variables retained in the model, including mixed/heterogeneous echogenicity, endomyometrial-junction abnormality, bright-edge sign, CDFI score, and polypoid mass-like morphology, are potentially operator-dependent. Although the examinations were performed by attending gynecologic sonographers using routine departmental reporting templates and the variables were interpreted according to IETA-style terminology, the retrospective design did not allow blinded central re-review or formal assessment of interobserver agreement. Misclassification of these predictors may therefore have occurred, particularly across the long study period and across different operators.

The exclusion of women with no qualifying endometrial or intracavitary lesion also limits generalizability. These women were not included because the model was designed to characterize risk among women with a detected lesion, and several predictors were lesion-level sonographic features that were not applicable in lesion-free patients. Therefore, the model should not be interpreted as a screening tool for all women undergoing pelvic ultrasound. Its intended use is risk stratification among women with histologically confirmed endometrial or intracavitary pathology. Including lesion-free women would require a different model structure, with lesion presence itself treated as a predictor and with separate handling of non-applicable lesion-level variables. Future studies should evaluate whether a broader two-stage model, first identifying the presence of a lesion and then estimating clinically significant pathology among lesion-positive women, performs better in unselected clinical populations.

Despite these limitations, the study offers several practical messages. First, endometrial thickness should not be interpreted alone once a lesion is visible or suspected; morphology and vascularity matter (2–5). Second, a bright-edge sign appears to remain a reassuring feature, but it should be interpreted within the broader lesion context (10, 15). Third, age and parity still matter, but their main value may be to trigger suspicion rather than to replace imaging-based assessment (8, 13). In practical terms, our results support a structured ultrasound-reading strategy in which thickness, lesion size, echogenicity, endomyometrial-junction status, and CDFI score are reported systematically and interpreted together with clinical characteristics (2, 10).

Future work should validate these findings prospectively and externally in independent cohorts. Prospective validation studies should incorporate standardized acquisition, predefined image-review protocols, and interobserver reproducibility assessment for the key sonographic predictors before broad clinical implementation. Also, further studies should also test whether more granular vascular-pattern descriptors, multi-reader image review, or molecular/histologic risk stratification improve performance without sacrificing interpretability. Comparison with newer AI-enabled ultrasound tools will also be important, but interpretable regression-based models are likely to remain valuable as transparent clinical benchmarks (11).

## Data Availability

The raw data supporting the conclusions of this article will be made available by the authors, without undue reservation.
