# Supplementary material for: Clinical and ultrasound predictors of clinically significant endometrial pathology in women with histologically confirmed lesions: a retrospective case–control study
Source: Front Oncol. 2026 Aug 26;16:1899603. doi: 10.3389/fonc.2026.1899603 (PMC13553436; doi:10.3389/fonc.2026.1899603)
Supplement: Supplementary file 1 [file Table1.docx]

**Supplementary Table S1. Pathology spectrum of the analytic cohort across benign pathology and clinically significant endometrial pathology.**

| **Final pathology category** | **Benign pathology (n=500)** | **Clinically significant pathology (n=500)** | **Overall (N=1000)** |
| --- | --- | --- | --- |
| Atrophy/normal | 239 (47.8%) | 0 (0.0%) | 239 (23.9%) |
| Polyp | 161 (32.2%) | 0 (0.0%) | 161 (16.1%) |
| Intracavitary myoma | 65 (13.0%) | 0 (0.0%) | 65 (6.5%) |
| Hyperplasia without atypia / endometritis / benign proliferative-secretory endometrium | 10 (2.0%) | 0 (0.0%) | 10 (1.0%) |
| EIN/atypical hyperplasia | 0 (0.0%) | 240 (48.0%) | 240 (24.0%) |
| Endometrial carcinoma | 0 (0.0%) | 260 (52.0%) | 260 (26.0%) |
| Other benign pathology | 25 (5.0%) | 0 (0.0%) | 25 (2.5%) |

**Supplementary Table S2. Sampling procedure type and ultrasound-to-pathology interval across benign pathology and clinically significant endometrial pathology.**

| **Variable** | **Overall (N=1000)** | **Benign pathology (n=500)** | **Clinically significant pathology (n=500)** | **P value** |
| --- | --- | --- | --- | --- |
| **Sampling procedure type, n (%)** |  |  |  | <0.001 |
| Hysteroscopic biopsy | 505 (50.5%) | 215 (43.0%) | 290 (58.0%) |  |
| D&C | 337 (33.7%) | 177 (35.4%) | 160 (32.0%) |  |
| Polypectomy/resection | 133 (13.3%) | 90 (18.0%) | 43 (8.6%) |  |
| Hysterectomy | 25 (2.5%) | 18 (3.6%) | 7 (1.4%) |  |
| Ultrasound-to-pathology interval, days | 34 (29–40) | 37 (31–43) | 32 (26–37) | <0.001 |

Continuous variables are shown as median (IQR) and categorical variables as n (%); P values represent between-group comparisons between women with benign pathology and women with clinically significant endometrial pathology. Continuous variable was compared using the Mann–Whitney U test; categorical variable was compared using the Pearson chi-square test or Fisher’s exact test, as appropriate; **Abbreviations**: D&C, dilation and curettage; EIN, endometrial intraepithelial neoplasia; IQR, interquartile range.

**Supplementary Table S3. Exploratory comparison of women with EIN/atypical hyperplasia versus endometrial carcinoma within the clinically significant pathology group.**

| **Variable** | **EIN/atypical hyperplasia (n=240)** | **Endometrial carcinoma (n=260)** | **P value** |
| --- | --- | --- | --- |
| Age, years | 61.0 (58.0–63.0) | 61.0 (58.0–63.0) | 0.581 |
| BMI, kg/m² | 23.3 (21.5–24.7) | 23.0 (21.7–24.5) | 0.690 |
| Endometrial thickness, mm | 12.0 (8.8–15.0) | 12.1 (8.7–15.4) | 0.783 |
| Lesion length, mm | 10.5 (9.6–11.7) | 10.6 (9.7–11.5) | 0.874 |
| Lesion volume, mL | 0.36 (0.30–0.43) | 0.36 (0.28–0.43) | 0.418 |
| Hemoglobin, g/dL | 13.3 (12.9–13.9) | 13.2 (12.7–13.6) | 0.014 |
| Postmenopausal status, yes | 227 (94.6%) | 247 (95.0%) | 0.843 |
| CDFI score moderate/abundant, yes | 86 (35.8%) | 85 (32.7%) | 0.509 |
| Polypoid mass-like lesion, yes | 138 (57.5%) | 143 (55.0%) | 0.589 |
| Family history of gynecologic cancer, yes | 33 (13.8%) | 26 (10.0%) | 0.213 |

Continuous variable was compared using the Mann–Whitney U test; categorical variable was compared using the Pearson chi-square test or Fisher’s exact, as appropriate; **Abbreviations:** EIN, endometrial intraepithelial neoplasia; IQR, interquartile range.

**Supplementary Table S4. Distribution of benign pathology and clinically significant pathology across calendar years**

| **Year** | **Benign pathology, n** | **Clinically significant pathology, n** | **Total, n** | **Clinically significant proportion** |
| --- | --- | --- | --- | --- |
| 2015 | 9 | 24 | 33 | 72.7% |
| 2016 | 11 | 26 | 37 | 70.3% |
| 2017 | 23 | 39 | 62 | 62.9% |
| 2018 | 16 | 48 | 64 | 75.0% |
| 2019 | 51 | 61 | 112 | 54.5% |
| 2020 | 44 | 72 | 116 | 62.1% |
| 2021 | 57 | 71 | 128 | 55.5% |
| 2022 | 68 | 48 | 116 | 41.4% |
| 2023 | 56 | 47 | 103 | 45.6% |
| 2024 | 60 | 20 | 80 | 25.0% |
| 2025 | 105 | 44 | 149 | 29.5% |

The case fraction varied significantly across years (Pearson chi-square P < 0.001), supporting the inclusion of a calendar-year sensitivity analysis.

**Supplementary Table S5. Sensitivity analyses of the combined clinicosonographic model.**

| **Analysis** | **N** | **Events, n (%)** | **Key adjusted findings** | **AUC** | **P value** |
| --- | --- | --- | --- | --- | --- |
| Calendar-year adjusted model (Added as a fixed-effect covariate) | 1000 | 500 (50.0) | Endometrial thickness remained significant: aOR 1.94 per 5 mm (95% CI 1.50–2.51; P<0.001). Bright-edge sign remained protective: aOR 0.37 (95% CI 0.20–0.68; P=0.002). | 0.966 | <0.001 |
| Excluding benign atrophy/normal histology | 761 | 500 (65.7) | Endometrial thickness remained significant: aOR 2.08 per 5 mm (95% CI 1.54–2.81; P<0.001). Bright-edge sign remained protective: aOR 0.31 (95% CI 0.16–0.62; P<0.001). | 0.959 | <0.001 |
| Postmenopausal women only | 705 | 474 (67.2) | Endometrial thickness remained significant: aOR 2.06 per 5 mm (95% CI 1.56–2.73; P<0.001). Polypoid mass-like lesion remained significant: aOR 3.77 (95% CI 2.22–6.38; P<0.001). | 0.941 | <0.001 |
| Carcinoma-only outcome (Redefined outcome as endometrial carcinoma only; EIN/atypical hyperplasia excluded) | 760 | 260 (34.2) | Endometrial thickness remained significant: aOR 2.07 per 5 mm (95% CI 1.54–2.77; P<0.001). Bright-edge sign remained protective: aOR 0.39 (95% CI 0.20–0.78; P=0.008). | 0.957 | <0.001 |
| Full eligible benign-cohort sensitivity analysis | 3271 | 500 (15.3) | Same combined model fitted using all 500 clinically significant cases and all 2771 eligible benign-pathology patients | 0.942 | <0.001 |

The same core predictor structure as the main combined model was retained whenever feasible: age, parity, endometrial thickness, lesion length, mixed/heterogeneous primary echogenicity, abnormal endomyometrial junction, CDFI score, bright-edge sign, and polypoid mass-like lesion. The sensitivity analysis was done using Multivariable binary logistic regression; likelihood-ratio test; ROC analysis; **Abbreviations:** aOR, adjusted odds ratio; AUC, area under the receiver operating characteristic curve; EIN, endometrial intraepithelial neoplasia.

**Supplementary Table S6. Normality assessment of continuous variables included in descriptive analyses.**

| **Variable** | **N** | **Shapiro–Wilk W** | **P value** |
| --- | --- | --- | --- |
| Age | 1000 | 0.985 | 1.98×10⁻⁸ |
| Parity | 1000 | 0.951 | 8.25×10⁻¹⁸ |
| BMI | 1000 | 0.987 | 1.31×10⁻⁷ |
| Hemoglobin | 1000 | 0.992 | 4.65×10⁻⁵ |
| Platelet count | 1000 | 0.920 | 1.55×10⁻²² |
| Endometrial thickness | 1000 | 0.973 | 1.44×10⁻¹² |
| Lesion length | 1000 | 0.988 | 1.60×10⁻⁷ |
| Lesion width | 1000 | 0.997 | 0.045 |
| Lesion anteroposterior diameter | 1000 | 0.996 | 0.0075 |
| Lesion volume | 1000 | 0.980 | 1.97×10⁻¹⁰ |
| Number of lesions | 1000 | 0.783 | 2.08×10⁻³⁴ |
| Ultrasound-to-pathology interval | 1000 | 0.995 | 0.0011 |

Normality was assessed using the Shapiro–Wilk test in the overall analytic sample. All listed variables showed statistically significant deviation from normality at P<0.05; therefore, continuous variables were summarized as median (IQR) and compared using the Mann–Whitney U test.
